# Randomized Clinical Trial on Ivermectin versus Thiabendazole for the Treatment of Strongyloidiasis

**DOI:** 10.1371/journal.pntd.0001254

**Published:** 2011-07-26

**Authors:** Zeno Bisoffi, Dora Buonfrate, Andrea Angheben, Marina Boscolo, Mariella Anselmi, Stefania Marocco, Geraldo Monteiro, Maria Gobbo, Giulia Bisoffi, Federico Gobbi

**Affiliations:** 1 Centre for Tropical Diseases, S. Cuore Hospital, Negrar (Verona), Italy; 2 Biostatistic Unit, University Hospital, Verona, Italy; George Washington University, United States of America

## Abstract

**Background:**

Strongyloidiasis may cause a life-threatening disease in immunosuppressed patients. This can only be prevented by effective cure of chronic infections. Direct parasitologic exams are not sensitive enough to prove cure if negative. We used an indirect immune fluorescent antibody test (IFAT) along with direct methods for patient inclusion and efficacy assessment.

**Methodology/Principal Findings:**

Prospective, randomized, open label, phase III trial conducted at the Centre for Tropical Diseases (Verona, Italy) to compare efficacy and safety of ivermectin (single dose, 200 µg/kg) and thiabendazole (two daily doses of 25 mg/Kg for two days) to cure strongyloidiasis. The first patient was recruited on 6^th^ December, 2004. Follow-up visit of the last patient was on 11^th^ January, 2007. Consenting patients responding to inclusion criteria were randomly assigned to one of the treatment arms. Primary outcome was: negative direct and indirect (IFAT) tests at follow-up (4 to 6 months after treatment) or subjects with negative direct test and drop of two or more IFAT titers. Considering 198 patients who concluded follow-up, efficacy was 56.6% for ivermectin and 52.2% for thiabendazole (p = 0.53). If the analysis is restricted to 92 patients with IFAT titer 80 or more before treatment (virtually 100% specific), efficacy would be 68.1% for ivermectin and 68.9% for thiabendazole (p = 0.93). Considering direct parasitological diagnosis only, efficacy would be 85.7% for ivermectin and 94.6% for thiabendazole (p = 0.21). In ivermectin arm, mild to moderate side effects were observed in 24/115 patients (20.9%), versus 79/108 (73.1%) in thiabendazole arm (p = 0.00).

**Conclusion:**

No significant difference in efficacy was observed, while side effects were far more frequent in thiabendazole arm. Ivermectin is the drug of choice, but efficacy of single dose is suboptimal. Different dose schedules should be assessed by future, larger studies.

**Trial Registration:**

Portal of Clinical Research with Medicines in Italy 2004&ndash;004693&ndash;87

## Author Summary

Strongyloidiasis is the infection caused by the worm *Strongyloides stercorali*s. Due to its peculiar life cycle *Strongyloides* may remain indefinitely in the host, if not effectively cured. Although the disease is usually mild, in case of weakening of the host's immune defenses the worm may invade virtually all organs and tissues (disseminated strongyloidiasis, almost invariably fatal). The treatment must then reach the goal of the complete elimination of the parasite. Small size clinical trials showed similar, high efficacy of the two drugs ivermectin (used as a single dose) and thiabendazole (used twice daily for two consecutive days). All trials used as the criterion for cure the absence of larvae in stool exams. The latter however may easily miss the infection, falsely suggesting that the infection has been cured. This trial, using a test detecting specific *Strongyloides* antibodies as an additional and more sensitive diagnostic tool, confirms previous reports: the two drugs have similar efficacy but ivermectin is better tolerated and is therefore the first choice. However the cure rate was lower than 70% for the standard, single dose. The authors then conclude that a larger, multi center trial is needed to find the optimal dose schedule of ivermectin.

## Introduction

Strongyloidiasis is a chronic, soil-transmitted infection caused by *Strongyloides stercoralis*, a helminth with a worldwide distribution, primarily in tropical and subtropical regions. Foci of low endemicity are also reported in temperate climates, such as the Mediterranean Coast, mostly among elderly patients [1], [2]. Prevalence data indicate that 30–100 million people are infected, but the figure is presumably underestimated [3].

Due to a peculiar life cycle that includes autoinfection (maintenance of parasitism in the absence of any further exposure to an external source), the infection can persist indefinitely, usually with mild and aspecific symptoms [3]–[6]. Nevertheless, disseminated strongyloidiasis, a life-threatening condition, may occur in case of immunosuppression [7], [8]. A suboptimal efficacy of the therapy of chronic strongyloidiasis may result in the persistence of the infection, with the potential risk of disseminated disease at any time. Several reported cases of fatal, disseminated disease had previously been treated and apparently cured [7], [9], [10].

Ivermectin is currently considered the best therapeutic option [11], [12]: trials comparing ivermectin and albendazole demonstrated unsatisfactory efficacy of the latter [13]–[16], while small sized trials comparing thiabendazole and ivermectin showed similar efficacy, but better tolerability of the latter [17], [18].

All trials conducted so far have exclusively relied on direct methods [17]–[22]. Therefore, the efficacy of any regimen could have been overestimated, because negative stool tests after treatment are no proof of eradication of the infection: the sensitivity of direct methods is largely unsatisfactory [3], [23], [24]. On the other hand, serology has been suggested as a reliable tool to monitor response to treatment [25]–[30].

This study was meant to compare the efficacy of ivermectin, administered as a single dose of 200 µg/kg, and thiabendazole, administered in two daily doses of 25 mg/Kg for two days, to cure strongyloidiasis.

## Methods

The protocol for this trial and supporting CONSORT checklist are available as supporting information: see Checklist S1 and Protocol S1.

### Study design and participants

This was a prospective, randomized, open label, phase III trial, carried out at the Centre for Tropical Diseases (CTD), Sacro Cuore Hospital, Negrar (Verona, Italy). Eligible patients were male and female subjects older than 5 years and weighing >15 kg, currently living in a non-endemic area; they had to have a diagnosis of strongyloidiasis established by indirect immune fluorescent antibody test (IFAT).

Exclusion criteria were: pregnancy or breastfeeding; CNS diseases; disseminated strongyloidiasis; immunodeficiency (malignancies, chemotherapy or other immunosuppressive treatments); planned travel to endemic countries before follow-up; lack of informed consent.

HIV positive subjects were excluded if CD4+ count was lower than 400/µl.

### Ethics

This research was conducted in full accordance to the Ethical Principles for Medical Research Involving Human Subjects as expressed in the Declaration of Helsinki and following amendments. Eligible patients were asked to meet the study investigator, who gave detailed explanation of the study protocol according to the patient information sheet and requested for written consent from the patient or, in case of minors, from her/his parent(s)/guardians. The study protocol was approved by the local Ethics Committee (Sacro Cuore Hospital Ethics Committee, 5^th^ August, 2004). All interventions (including unscheduled visits) were at no charge to the patients.

### Interventions

Potentially eligible subjects attending the study clinic were identified through laboratory diagnosis of *S. stercoralis* infection as defined above. Indirect immune fluorescent antibody test (IFAT) was performed in accordance with the procedures described in detail elsewhere [29]. Stool agar plate culture and microscopic examination (after concentration according to Ritchie) were performed if not previously available. Baseline assessment also included routine haematology with WBC differential count and routine chemistry.

Consenting patients were admitted to the clinic for at least three days for a close monitoring of side effects; on admission a Case Report Form (CRF) was filled with the patient's unique ID number. Clinical examination and history were carried out on admission, according to the CRF. Based on the randomization list, patients were given either ivermectin or thiabendazole. Ivermectin (tablets 3 mg) was administered at the single dose of 200 µg/kg on an empty stomach, and patients were instructed to keep fasting for the following 2 hours. Thiabendazole (tablets 600 mg) was administered with food, in two daily doses of 25 mg/Kg for two days. The drug intake was directly observed by a nurse.

The patients were asked to attend the clinic twice after treatment completion: after one month and after four months. At both follow-up visits, clinical history and examination were carried out and a full blood count (FBC) was performed. At the second visit only, IFAT was performed, and so was a stool agar plate culture (if positive on recruitment). As was the routine procedure at CTD laboratory, follow-up serum samples were tested in parallel with those of the initial diagnosis. If the patient did not present for the second follow-up visit, the investigator had to contact her/him and fix another appointment. The second follow-up visit, at which the efficacy outcomes were assessed, was considered still valid up to 6 months from the treatment. Patients who did not present within the 6 months were considered lost to follow-up.

### Objectives

Primary objective was to compare the efficacy of ivermectin, administered as a single dose of 200 µg/kg, and thiabendazole, administered in two daily doses of 25 mg/Kg for two days, to cure strongyloidiasis. Secondary objective was to assess safety and tolerability of both regimens.

### Outcomes

Primary outcome was cure at Time 2 (T2: 4 to 6 months after recruitment), defined as follows: negative stool agar culture for *S. stercoralis* (assessed in case of positivity of any direct stool tests on recruitment), AND: negative IFAT or decrease of two or more antibody titers. Secondary outcome was: patients with adverse reactions (grade 1 to 5 as defined below) to treatment.

All adverse events reported by the patients on days 1 and 2 of treatment were recorded in the patient's CRF, and so were adverse events recorded during scheduled and unscheduled visits. Adverse events for this study purpose were graded as: 0 = None; 1 = Mild: any symptoms possibly related to drug, not necessitating medication; 2 = Moderate: any symptoms possibly related to drug, requiring medication; 3 = Serious: requiring treatment to be discontinued; 4 = Near fatal: requiring intensive care; 5 = Fatal.

### Sample size

The sample size was determined based on the primary outcome. The trial was designed to detect a difference of efficacy of at least 15% with a study power of 80% and p<0.05 for alternative hypotheses, 2-sided and with a minimal efficacy of 70% for the less effective regimen: the required sample size was of 133 subjects in each group. Considering subjects lost to follow-up, a total of 150 patients for each treatment group was initially planned to be enrolled.

### Randomization

Subjects were randomly assigned to one of the following arms with allocation ratio 1∶1. Group A: ivermectin 200 µg/kg in a single dose. Group B: thiabendazole, 25 mg/Kg b.i.d for two days. The randomization list was computer-generated by a biostatistician who was not directly involved in any other operational aspect of the study and handed to the nurse in charge, who was not directly involved in the study, either, and kept the list in a locked drawer. When a patient was considered to meet the inclusion criteria and had given her/his informed consent, the patient was formally recruited by the study investigator (ZB, AA, GM, MA, MB or SM) who was on duty, who then reported the patient's unique ID number and the general data in the CRF. The nurse in charge (or her delegate in her absence) was then asked to indicate the allocation group according to the ID number and treatment was started immediately. As randomization was not in blocks, there was no way for the investigator to guess in advance what the next assigned treatment would be. More rigorous procedures (such as the use of sealed envelopes labelled with the unique ID number and containing the indication of allocation) were not judged necessary.

### Blinding

This was an open label trial that exclusively relied on lab values for the assessment of the primary outcome, therefore blinding of laboratory staff was ensured: the laboratory personnel performing the analyses (stool culture, serology) had no direct contact with the investigators and no information as regards the drug administered to the patient.

### Statistical methods

Data were double entered with Epi Info software (CDC Atlanta, version 3.3.2) and analysed with the same software and with Stata 9.2 (StataCorp LP, College Station, TX 77845 USA). The two randomised groups were first compared with respect to baseline demographic and clinical data. Proportions were compared through Yates' chi-square test. T test for independent groups was used for continuous variables. Mann-Whitney U test was used for non normal variables. The pattern of compliance to treatment and to follow-up visits was also explored and compliers/non compliers were compared with respect to baseline data. Patients with missing values and patients lost during treatment or at follow-up were to contribute to the analysis only for the time during which data were available.

The analysis of primary as well as secondary outcomes was planned on an intention-to-treat basis (ITT) considering all subjects as originally assigned to the two arms. As all patients were able to conclude their treatment according to plan, and as we subsequently excluded from the analysis of efficacy patients lost to follow-up whose outcome was unknown, this corresponded, de facto, to a per-protocol (PP) analysis [31].

The proportions of patients with the occurrence of the binary, primary and secondary outcomes of interest (as defined above) in each of the two arms were compared through the Yates' chi-square test with continuity correction. Fisher's exact test was used when appropriate. No subgroup analysis was initially planned. Subsequently however, a separate analysis was carried out on subgroups, in order to be able to better compare our results with those of previous trials based on direct diagnostic criteria only.

## Results

The study started with the recruitment of the first patient on the 6^th^ December 2004, while the last one was recruited on the 3^rd^ August 2006. At that moment, 223 patients had been included in the study. Recruitment was concluded before the required sample size was attained. The reason was the obvious difference in tolerability observed by the investigators between the two arms. Although this was not an explicitly defined criterion for the early conclusion of the study (as all observed side effects were mild to moderate), the recruitment was interrupted and an interim analysis was carried out in November, 2006, on the 187 patients who had concluded follow-up. The analysis showed a very similar cure rate between the two arms, while the frequency of side effects was much higher in the thiabendazole arm. Then, on 27^th^ November, 2006, the decision to stop recruitment was notified to the Ethical Review Board. After that date, 11 more patients, previously recruited, presented to follow-up until January, 2007, when the data lock occurred, after the second follow-up visit of the last patient (11^th^January, 2007), therefore the final analysis of the primary endpoint concerned 198 subjects.

### Participant flow

**Figure 1:**
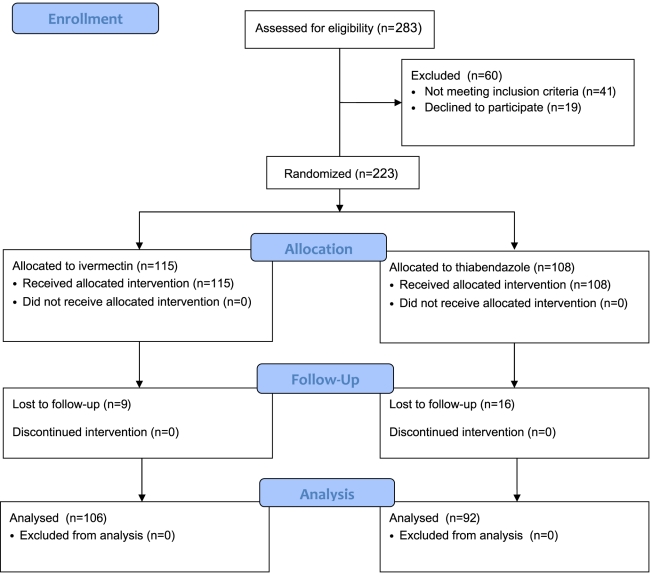
CONSORT flow diagram. The flow of patients through the randomized clinical trial.

The flow of patients assessment and enrollment is reported in the study flow diagram (Figure 1). Briefly, out of 283 patients initially screened for inclusion, 242 were eligible for inclusion, of whom 223 gave their written (or their guardians') informed consent and were recruited. Of the patients recruited, 115 (51.6%) were randomly assigned to ivermectin arm and 108 (48.4%) to thiabendazole. Follow-up was completed by 198 patients (88.8%), 106 (53.5%) assigned to ivermectin and 92 (46.5%) to thiabendazole.

### Baseline data

The main baseline characteristics of the randomized population (223 patients) is reported in Table 1. None of the observed differences between the two groups was statistically significant.

**Table 1:** Comparison of randomized groups.

*Main characteristics*	*Ivermectin*	*Thiabendazole*	*p*
Sex M	70 (60.9%)	61 (56.5%)	0.60
Age (Years) (Mean)	43.9 (SD 20.9)	41.3 (SD 19.3)	0.29
Weight (Mean)	68.5 (SD 18.0)	66.5 (SD 16.3)	0.38
Prior residence overseas	29/115 (25.2%)	22/108 (20.4%)	0.41
Symptomatic	50/115 (43.4%)	54/108 (50.0%)	0.71
Eosinophils/mmc (Mean)	718 (SD 819)	852 (SD 1141)	0.26§
IFAT titer (Median)	40 (Q1 = 20, Q3 = 160)	60 (Q1 = 40, Q3 = 80)	0.89§
Positive stool agar culture	35/79 (44.3%)	27/79 (34.2%)	0.22
Positive stool microscopy	34/103 (33.0%)	35/96 (36.5%)	0.71

Inference on proportions based on Yates' chi-square test, inference on continuous variables based on Student's T test, unless otherwise specified.

§Mann-Whitney U test.

In the following table the baseline characteristics of the patients ultimately analysed for efficacy are compared with those of patients lost to follow-up (Table 2). All patients lost to follow-up (25/25) belonged to the group of “residents overseas”, which included immigrants. Compliance to follow-up was higher for ivermectin (106/115 or 92.2%) than thiabendazole (92/108 or 85.2%), but the difference was not statistically significant (p = 0.15).

**Table 2:** Comparison of baseline characteristics of patients who completed / did not complete follow-up.

*Main characteristics*	*Follow-up completed (n. 198)*	*Lost to follow-up (n. 25)*	*p*
Sex M	115 (87.8%)	16 (12.2%)	0.72
Age (Years) (Mean)	43.9 (SD 20.7)	31.8 (SD 9.8)	0.01
Weight (Mean)	67.6 (SD 17.4)	67.3 (SD 15.4)	0.99
Prior residence overseas	147/198 (74.2%)	25/25 (100%)	0.00
Symptomatic	92/198 (46.5%)	12/25 (48%)	0.95
Eosinophils/mmc (Mean)	777 (SD 609)	835 (SD 1030)	0.11§
IFAT titer (Median)	40 (Q1 = 20, Q2 = 160)	(80 Q1 = 40, Q2 = 160)	0.27§
Positive stool agar culture	85/198 (42.9%)	8/25 (32.0%)	0.69
Positive stool microscopy	61/198 (30.8%)	8/25 (32.0%)	0.91

Inference on proportions based on Yates' chi-square test, inference on continuous variables based on Student's T test, unless otherwise specified.

§Mann-Whitney U test.

### Efficacy

We first assessed the efficacy on all 198 subjects included (Table 3) who were assessed at follow-up. Based on the primary endpoint (all criteria fulfilled), the subjects cured were 60/106 (56.6%) and 48/92 (52.2%) in ivermectin and thiabendazole arm, respectively (p = 0.53). If we considered as cured, with less stringent criteria, also the subjects with a partial response (negative stool culture and decrease of only one IFAT titer), efficacy would rise to 75/106 (70.8%) and to 67/92 (72.8%), respectively (p = 0.75). If we considered as criteria of cure the direct methods only (negative stool culture at follow-up in subjects who were positive at microscopy and/or culture on recruitment), efficacy would be 30/35 (85.7%) and 35/37 (94.6%), respectively (p = 0.21). We then did the same analyses on the subgroup with IFAT titer ≥80 (virtually giving no false positive results) on recruitment (Table 4). On this sub group, comprising about half the total sample (92 subjects), all criteria were fulfilled by 32/47 subjects (68.1%) in ivermectin arm and 31/45 (68.9%) in thiabendazole arm (p = 0.93). Including subjects with a partial response as defined above, efficacy would be 41/47 (87.2%) and 40/45 (88.9%), respectively (p = 0.81). Finally, considering direct methods only in this subgroup, the cure rate would be 22/24 (91.7%) and 27/27 (100%), respectively (p = 0.22).

**Table 3:** Outcome at follow-up (month 3^rd^–6^th^) in the two arms (198 patients who concluded follow-up).

Measures of efficacy	*Ivermectin*	*Thiabendazole*	*p*
All criteria fulfilled	60/106 (56.6%)	48/92 (52.2%)	0.53
Patients with partial response^1^ included	75/106 (70.8%)	67/92 (72.8%)	0.75
Efficacy based on negativization of stool microscopy and/or culture	30/35 (85.7%)	35/37 (94.6%)	0.19∧

Inference on proportions based on Yates' chi-square test, inference on continuous variables based on Student's T test, unless otherwise specified.

**∧:** Fisher's exact test.

1 Patients with negative stool and drop of only one antibody titer.

**Table 4:** Outcome at follow-up (month 3^rd^–6^th^) in the two arms (92 patients with IFAT titer> = 80).

Measures of efficacy	*Ivermectin*	*Thiabendazole*	*p*
All criteria fulfilled	32/47 (68.1%)	31/45 (68.9%)	0.93
Patients with partial response^1^ included	41/47 (87.2%)	40/45 (88.9%)	0.81
Efficacy based on negativization of stool microscopy and/or culture	22/24 (91.7%)	27/27 (100%)	0.22∧

Inference on proportions based on Yates' chi-square test, inference on continuous variables based on Student's T test, unless otherwise specified.

**∧:** Fisher's exact test.

1 Patients with negative stool and drop of only one antibody titer.

### Adverse events

As side effects of the two drugs are known to be limited in time, we considered for this outcome all 223 patients included and not only those who completed the follow-up. No serious side effect (grade 3 or more) was observed in any patient. Overall, 103/223 patients complained of any side effect, grade 1 to 2 (Table 5). In ivermectin arm, side effects were observed in 24/115 patients (20.9%), versus 79/108 (73.1%) in thiabendazole arm (p = 0.00). Only 5/115 (4.3%) patients in the ivermectin arm presented effects of grade 2 (requiring medication), while in thiabendazole arm 43/108 patients (39.8) presented effects of grade 2 (p = 0.00). Dizziness was the most frequently reported side effect both in thiabendazole arm (57/79 or 72.2%, followed by nausea and vomiting) and in ivermectin arm (12/24 or 50.0%, followed by day somnolence) (data not reported in tables).

**Table 5:** Side effects (analysis on all 223 patients included in trial).

Side effects	*Ivermectin*	*Thiabendazole*	*p*
Grade 1	19/115 (16.5%)	36/108 (33.3%)	0.01
Grade 2	5/115 (4.3%)	43/108 (39.8%)	0.00
All	24/115 (20.9%)	79/108 (73.1%)	0.00

Inference on proportions based on Yates' chi-square test, inference on continuous variables based on Student's T test, unless otherwise specified.

## Discussion

### Interpretation

This was the first trial on strongyloidiasis treatment using serology along with direct methods for case inclusion and assessment of efficacy. The latter, based on primary outcome, was lower than 60%, with no significant difference between the two treatment arms. With less strict criteria (including partial response as defined above), efficacy would rise to above 70% for both regimens, still with no significant difference. As the specificity of IFAT, though very high, is not 100% for the lower dilutions [29], the inclusion of some false positives may have occurred and partly explain the low efficacy found. We then analyzed a sub group of patients who had a serologic titer ≥80 (virtually giving no false positive results) [29] on recruitment. Efficacy as defined by primary outcome, and efficacy including partial response as defined above, were significantly higher in this subgroup for both regimens (close to 70% and to 90%, respectively), suggesting a more correct case inclusion. Finally, when we analysed only the patients who had positive stool tests on inclusion, taking culture negativization as a criterion for cure, the efficacy was close to or higher than 90% for both regimens, approaching that found by other studies [15], [17]–[19], [22]. Thus, the lower efficacy found by our study is clearly due to the more strict criteria used to define cure, that include serology. Our data confirm that serology tends to decrease in titer within a few months of effective treatment and can thus be a useful tool for treatment monitoring as was previously suggested [25]–[30]. Considering subjects with serologic titer ≥80 on inclusion, almost 90% had a drop of titer following treatment.

### Overall evidence

Whatever the criterion used, we were not able to find any significant difference between thiabendazole and ivermectin at standard dose for the cure of *S. stercoralis* infection. This finding confirms previous, smaller trials [17], [18].

Both drugs appeared to be safe, with no serious side effect in either treatment arm. Nevertheless, thiabendazole caused significantly more side effects and of higher grade.

### Study limitations

This trial was not double blind. This cannot have affected the assessment of efficacy, as the primary outcome was entirely based on laboratory investigations and lab staff was kept unaware of the treatments administered. Contrarily, side effect reporting might have been influenced both by the investigator's and the patient's knowledge of the drug received. Results are therefore to be taken with some caution, though the difference between the two arms was clearly too big to be entirely attributable to bias.

Inclusion criteria allowed the recruitment of patients with negative direct tests on stool and positive serology at any IFAT titer. As discussed above, this probably caused some patients without the infection to be erroneously included with a consequent underestimation of the efficacy of both drugs. Subsequent analysis showed that more strict criteria (based on a minimal required cutoff of dilution) should be followed for trial inclusion. We believe the analysis of the subgroup of subjects with IFAT titer ≥80 to provide the more reliable estimate of efficacy. Given this more strict inclusion criteria, the analysis still failed to show any significant difference between the two regimens, but the sample size was originally calculated only to detect a 15% difference between the original groups.

### Future research

Finally, we still remain with the problem of the lack of a gold standard to define cure. We think that serology should also have a role at least in a scenario like ours, with most probably no more local transmission, where the interpretation of the results is not potentially confounded by reinfection. While awaiting alternative diagnostic methods such as real time PCR [32] to become a reliable alternative, the best option is probably the combination of direct with indirect methods, but the latter need further study to identify the optimal serologic test and cutoff for diagnosis, trial inclusion and treatment follow-up.

Ivermectin is the treatment of choice due to better tolerability, but the single dose efficacy is sub optimal. Some guidelines and the WHO drug formulary have already shifted to a new schedule (200 µg/Kg/day for two consecutive days) [11], [12], while some experts recommend the repetition of treatment after two weeks, on ground of the parasite life cycle [33]. Though it seems reasonable to expect that the use of an increased dose would improve the efficacy of ivermectin, neither of these alternative regimens has ever been validated by a randomized trial to our knowledge, and the last published trial [13] failed to show any significant difference between the single dose and two doses two weeks apart.

Considering that no truly promising new drug is in the pipeline, we are planning a multi center trial on different dose schedules of ivermectin.

## Supporting Information

Checklist S1 CONSORT Checklist.
(DOC)

Protocol S1 Trial Protocol.
(DOC)
