# Supplementary material for: Randomized Clinical Trial on Ivermectin versus Thiabendazole for the Treatment of Strongyloidiasis
Source: PLoS Negl Trop Dis. 2011 Jul 26;5(7):e1254. doi: 10.1371/journal.pntd.0001254 (PMC3144183; doi:10.1371/journal.pntd.0001254)
Supplement: Protocol S1 — Trial Protocol. (DOC) [file pntd.0001254.s002.doc]

**Studio randomizzato di terapia dell’infestazione da *Strongyloides stercoralis*: confronto di ivermectina vs thiabendazolo**

**protocollo operativo**

**INDICE**

Sintesi 4

Reclutamento 5

Consenso informato (modulo 1) 6

Informazioni al paziente (modulo 2) 6

Indagini diagnostiche 6

Trattamento 6

Randomizzazione e registrazione del paziente 7

Eventi avversi 7

Follow-up a 4 e 12 mesi 7

Flow chart 1 8

Analisi statistica 8

SINTESI

Reclutamento

1. Verifico se il paziente risponde ai criteri di inclusione
2. Verifico l’assenza di criteri di esclusione
3. Propongo lo studio: stampo e spiego al paziente i moduli 1 (Consenso Informato) e 2 (Informazioni per il paziente); li firmo e li faccio firmare al paziente (o al suo tutore legale o testimone)

Indagini diagnostiche

Faccio eseguire al paziente gli esami diagnostici non ancora eseguiti: TUTTI i pazienti DEVONO ESEGUIRE:

- Emocromo con formula, AST, ALT
- Esame coproparassitologico (su 3 campioni) e coprocoltura in agar presso questo Centro
- Sierologia per *Strongyloides* IFAT

Eseguo inoltre:

- sierologia per HIV e, se donna in età fertile, test di gravidanza

Registrazione e Scheda Clinica

1. Registro data di reclutamento, nome e peso corporeo del paziente sulla lista di randomizzazione, assegnandogli un codice (numero progressivo)
2. Prendo la cartella ambulatoriale dello studio e registro i dati del paziente, il codice, il numero di telefono (meglio se fisso e mobile), i dati del Medico Curante (nome e telefono)
3. Stampo e compilo la Scheda Clinica (Modulo 3)
4. Programmo il giorno del ricovero (in modo che abbia già fatto tutti gli esami) e lo segno sulla cartella

Randomizzazione

1. La lista di randomizzazione viene conservata della caposala in armadio chiuso a chiave.
2. Ad ogni arruolamento, chiedere alla caposala (o sua delegata, se assente) di verificare il braccio nel quale ricade il paziente, e trattarlo di conseguenza.

Trattamento

1. Il giorno 1 prestabilito accolgo il paziente, misuro la PA e la registro sulla scheda clinica
2. Somministro il trattamento previsto per il giorno 1
3. Misuro la PA almeno 3 volte nelle 8 ore successive
4. Il giorno 2 ripeto la procedura dei punti 2 e 3 per il tiabendazolo
5. Programmo le date dei due follow-up e avviso il paziente che sarà richiamato

Follow-up a 1 e a 4 mesi

1. Chiamo il paziente qualche giorno prima per ricordargli l’appuntamento
2. Alla prima visita di follow-up eseguo prelievo per emocromo con formula. Valutazione clinica (compilo la Scheda Clinica).
3. Alla seconda visita di follow up eseguo prelievo per emocromo con formula, IFAT, esame coproparassitologico diretto, coprocoltura in agar. Valutazione clinica (compilo la Scheda Clinica).

Reclutamento

Il Laboratorio segnala all’Investigator la positività di un paziente per un esame sierologico o parassitologico diretto (coproparassitologico con arricchimento secondo Ritchie o coprocoltura in agar) per *S. stercoralis*. Se la diagnosi è stata eseguita esclusivamente con una metodica diretta, è necessario che venga eseguita anche la sierologia, che deve risultare positiva per poter arruolare il paziente.

Se il paziente risulta positivo all’IFAT, verrà subito affidato alle cure del responsabile dello studio, che verificherà la presenza dei seguenti criteri di inclusione/esclusione:

- **Criteri di inclusione**:
- Positività all’IFAT per *Strongyloides* > 1:20
- Età > 5 anni, peso > 15 Kg
- Residenza attuale in area non endemica e non programmi di spostamenti in area endemica prima della fine del follow up
- **Criteri di esclusione**:
- gravidanza o allattamento
- diagnosi di strongiloidiasi disseminata
- patologie gravi del SNC
- malattie onco-ematologiche, trattamenti anti-blastici e/o immunosoppressivi in corso
- linfociti CD4+ < 400/μl (per i soggetti HIV positivi)
- Presenza di microfilarie di *Loa loa* nel sangue periferico.

Se i criteri sono soddisfatti, l’investigator propone lo studio al paziente e gli fissa un appuntamento per presentarsi al Centro per ricevere il trattamento (giorno 1).

Consenso informato (modulo 1)

Il medico responsabile dello studio presso il Centro propone al paziente la partecipazione allo studio. Lo informa in modo comprensibile, completo e imparziale della possibilità di aderire volontariamente alla sperimentazione in forma anonima. Al paziente vengono spiegati in modo esaustivo potenziali rischi, benefici, durata della partecipazione e possibili alternative. Gli viene inoltre spiegato che in qualsiasi momento potrà decidere di interrompere questa partecipazione. Ottiene quindi dal paziente un consenso scritto, mediante la firma sull’apposito modulo di consenso informato. In caso di minore, la firma viene apposta da un genitore o da persona che ne esercita la patria potestà. In caso di soggetto non in grado di leggere, la firma viene apposta da un testimone imparziale che avrà assistito alla fornitura di adeguate informazioni al paziente al consenso orale dello stesso.

Informazioni al paziente (modulo 2)

Vengono consegnate al paziente informazioni scritte aggiuntive sulla strongiloidiasi e sullo studio a cui ha deciso di aderire, disponibili in italiano o in inglese, che verranno firmate dal medico sperimentatore e da lui stesso.

Indagini diagnostiche

Ogni paziente rispondente ai criteri di inclusione, deve eseguire almeno una routine ematochimica (comprendente almeno emocromo con formula, AST, ALT), se non già eseguita negli ultimi 30 giorni. Occorre inoltre verificare che il paziente abbia eseguito esame coproparassitologico su almeno 3 campioni, con arricchimento secondo Ritchie, e una coprocoltura in agar per *S. stercoralis.* Soggetti provenienti da paesi endemici per *Loa loa* verranno sottoposti a ricerca delle microfilarie mediante leucoconcentrazione di 10 cc. di sangue.

## Trattamento

Il giorno 1 il paziente viene accolto in reparto in regime di ricovero ordinario. Si esegue una nuova verifica sul fatto che il paziente risponda ai criteri di inclusione/esclusione. Si richiede quindi alla caposala di verificare in quale braccio di randomizzazione (vedi paragrafo successivo) si deve collocare il paziente. Deve quindi essere compilata una Scheda Clinica (Modulo 3) dello studio con i dati e il codice del nuovo paziente. Se, in relazione alla lista di randomizzazione, il paziente deve ricevere ivermectina, rimane a digiuno da almeno 6 ore. Gli viene misurata la pressione arteriosa, che viene registrata sulla scheda clinica. Se donna in età fertile, si deve controllare che sia stata esclusa una gravidanza in corso. Gli viene quindi somministrato il farmaco e il paziente rimane a digiuno per altre 2 ore. Se il paziente deve assumere tiabendazolo, questo farmaco gli viene somministrato dopo la colazione e dopo la cena, nel giorno 1 e 2.

Vengono effettuate almeno 3 misurazioni di pressione arteriosa nelle 8 ore successive al trattamento e i valori vengono registrati sulla CRF del paziente.

Al termine del trattamento viene programmata la prima data di follow-up (a 1 mese) e si consegna un promemoria al paziente, avvisandolo che sarà richiamato.

Randomizzazione e registrazione del paziente

Il paziente deve essere registrato con nome e cognome sulla **lista di randomizzazione** con i codici dei pazienti in progressione, la data di reclutamento e il peso corporeo. Tale lista, generata dal biostatistico, verrà custodita in un cassetto chiuso a chiave nella stanza della caposala, che non verrà coinvolta nello studio per altri aspetti. Non saranno impiegate ulteriori procedure più rigorose per mantenere il riserbo riguardo la lista di randomizzazione, in quanto si tratta di uno studio spontaneo, non sponsorizzato, e nessun investigator ha motivi per “forzare” l’arruolamento di un paziente in un braccio piuttosto che in un altro.

Eventi avversi

Il secondo obiettivo principale dello studio è il confronto della tollerabilità dei due regimi terapeutici. Gli outcomes secondari dello studio sono pertanto gli eventuali eventi avversi che si verificheranno durante il ricovero e che verranno registrati nella scheda paziente secondo il seguente grading:

1 Lieve: non ha richiesto trattamento;

2 Moderato: ha richiesto trattamento;

3 Grave: ha richiesto interruzione del trattamento;

4 Emergenza clinica: ha richiesto cure intensive.

5 Fatale.

In realtà entrambi i farmaci sono stati somministrati a migliaia di pazienti e sono considerati in genere ben tollerati. Gli unici (rari) casi di effetti avversi gravi da ivermectina sono stati segnalati in soggetti con infezione da *Loa loa* ad alta carica. Soggetti risultati positivi per *Loa loa* verranno esclusi dallo studio.

Follow-up a 1 e 4 mesi

Il paziente viene chiamato il giorno precedente il controllo programmato. Il giorno del controllo viene eseguito un prelievo per emocromo con formula e vengono valutati segni e sintomi, che venogno registrati sulla CRF. Alla seconda visita di follow up vengono inoltre eseguiti sierologia IFAT *Strongyloides* e test diretti se positivi alla diagnosi.

### Flow chart 1: schematizzazione delle principali procedure da eseguire prima del trattamento

Comunicazione dal laboratorio di esame positivo per *Strongyloides*

IFAT positivo: paziente arruolabile

Diagnosi eseguita con esame diretto: eseguire IFAT

IFAT negativo: paziente escluso

Verificare criteri di inclusione/esclusione. Programmare eventuali esami mancanti

Consegnare moduli 1 e 2 al paziente. Fissare appuntamento per il trattamento

Giorno 1: nuova verifica dei criteri di inclusione/esclusione

Consegna da parte del paziente del consenso informato firmato

Verifica del braccio di randomizzazione

Trattamento

Analisi statistica

I dati verranno inseriti da due diversi investigatori in un database creato con il software Epi Info. Gli eventuali record discordanti vengono rivisti da un terzo investigatore.

I dati verranno analizzati usando lo stesso Epi Info e Stata.

I due gruppi verranno inizialmente confrontati rispetto ai dati generali (baseline) demografici e clinici. Si esplorerà il pattern di compliance al trattamento e al follow-up e i due gruppi di pazienti compliants – non compliants al follow-up saranno confrontati rispetto alle principali variabili e al trattamento assegnato.

L’analisi degli outcome primari e secondari sarà su base intention-to-treat (ITT). Essendo molto breve la durata del trattamento è improbabile che dei pazienti passino al trattamento alternativo nel corso del trial, ma potrebbe succedere, in teoria, per il solo thiabendazolo (l’ivermectina prevede un’unica somministrazione) ad esempio per insorgenza di effetti collaterali che inducano a cambiare farmaco. Sempre in teoria, potrebbe inoltre succedere che i pazienti vengano trattati nuovamente con lo stesso farmaco, o con il farmaco alternativo, prima del follow-up. Se queste situazioni si dovessero effettivamente verificare, anche un’analisi per-protocol (PP) sarà eseguita, includendo solo i pazienti che avranno eseguito il trattamento come specificato nel protocollo.

Le proporzioni saranno confrontate tramite Yates’ chi-square test. Il Fisher’s exact test verrà utilizzato ove appropriato (effettivo inferiore a 5 in almeno una cella). Il T test di Student sarà utilizzato per variabili continue, il Mann-Whitney U test per variabili non normali.

Non sono previste analisi per sottogruppi.
